# Multitargeted anti-infective drugs: resilience to resistance in the antimicrobial resistance era

**DOI:** 10.4155/fdd-2022-0001

**Published:** 2022-05-05

**Authors:** Colin J Suckling, Iain S Hunter, Fraser J Scott

**Affiliations:** 1 Department of Pure & Applied Chemistry, University of Strathclyde, 295 Cathedral Street, Glasgow, G1 1XL, UK; 2 Strathclyde Institute of Pharmacy & Biomedical Sciences, University of Strathclyde, 161 Cathedral Street, Glasgow, G4 0RE, UK

**Keywords:** anti-infectives, antimicrobial resistance, DNA, multitargeting anti-infective drugs, nucleic acids, RNA, Strathclyde minor groove binders, strathclyde nucleic acid binders

## Abstract

The standard drug discovery paradigm of single molecule – single biological target – single biological effect is perhaps particularly unsuitable for anti-infective drug discovery. This is due to the rapid evolution of resistance likely to be observed with single target drugs. Multitargeted anti-infective drugs are likely to be superior due to their lower susceptibility to target-related resistance mechanisms. Strathclyde minor groove binders are a class of compounds which have been developed by adopting the multitargeted anti-infective drugs paradigm, and their effectiveness against a wide range of pathogenic organisms is discussed. The renaming of this class to Strathclyde nucleic acid binders is also presented due to their likely targets including both DNA and RNA.

Even before the COVID-19 pandemic it was well understood scientifically and politically that the world badly needs new anti-infective drugs to tackle bacterial, fungal, parasitic and viral infections [1]. The emergence of resistant strains of all types of pathogens has led to untreatable diseases worldwide, which have been categorized by the WHO [2]. Calls from scientists and policy makers to take new scientific and commercial approaches to anti-infective therapy have become common [3,4]. Scientifically, resistance arises from a number of mechanisms of which mutation of the drug target (protein or rRNA) is most relevant here. It is obvious that a drug that engages with more than one biological target should be less susceptible to the emergence of resistance by this mechanism, hence the significance of multitargeted anti-infective drugs (MTAIDs)*.*

A further challenge for new anti-infective drugs is that the current commercial opportunities for anti-infective compounds in general are commonly regarded as unfavorable by major pharmaceutical companies in comparison with other therapeutic fields. Factors that contribute to this view are the risk of early obsolescence of a new drug because of the development of resistance combined with reluctance to use in the clinic to minimize the development of resistance both of which tend to limit sales. With both the limited discovery research in anti-infective medicines and the need for an approach that intrinsically minimizes the development of resistance, scientists at the University of Strathclyde have developed the so-called Strathclyde minor groove binders (S-MGB) platform, of novel compounds that act at multiple cellular targets within an organism. As will be described below, this research has led to a new antibacterial drug for the treatment of *Clostridioides difficile* infections that has successfully completed a Phase IIa clinical trial and is ready for Phase III [5] and, using other members of the platform, to the demonstration of effectiveness in animal models of infection of fungal, parasitic and viral infections.

The idealized standard industrial drug discovery paradigm is single drug molecule – single biological target – single biological effect (Figure 1). In contrast, S-MGB research took a different approach and looked for compounds that might have multiple parallel effects on the pathogen; thereby, reducing the risk of rapid evolution of resistance in the clinic, in other words, MTAIDs (Figure 1). All anti-infective drugs can be expected to lead to resistant strains but the standard drug discovery paradigm is in a sense primed for the development of resistance because, for example, a single amino-acid change in the target protein could directly cause resistance to the new drug. Many current clinical trials for anti-infective drugs are studying combination therapies [6] and there are increasingly examples being published of new drugs in other fields designed to have more than one target, mostly from academic groups where speculative approaches to new drugs can be easily justified [7]. Multitargeting in the context of anti-infective drugs is undoubtedly becoming more widely investigated [8,9]. This is not to suggest that single targeted drugs are no longer of interest. There are many examples in antibacterial, antifungal, antiparasistic and antiviral fields [10–13].

**Figure 1.**
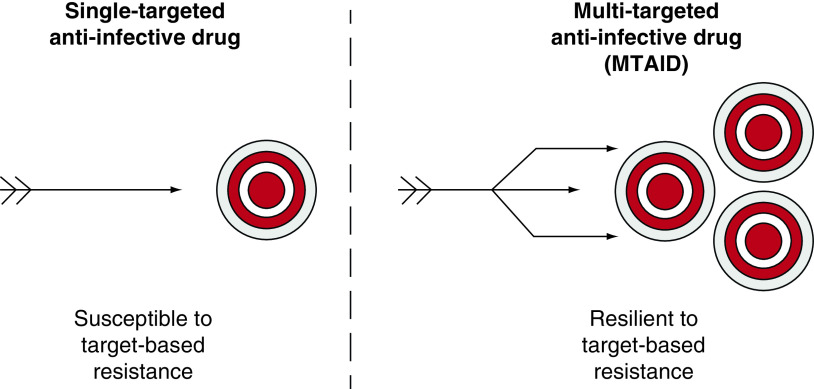
Single-targeted and multitargeted anti-infective drugs.

The increasing interest in multitargeting notwithstanding, it is to be expected that even a combination of single molecular targets will lose efficacy if one essential component is mutated to abrogate drug binding. However, for drugs that target the minor groove of DNA in a non selective way (i.e., can engage with multiple specific base-pair sequences), the substantial number of potential binding sites within the minor groove makes inactivation through a single mutation very improbable. Provided that safety can be obtained, DNA and DNA centric processes make very relevant targets for anti-infective therapy in the AMR era. In this context, the S-MGB platform is built upon the natural products, distamycin and netropsin, that have been known as anti-infective compounds since the 1960s (Figure 2) [14,15]; the platform takes the efficacy and scope of these prototype natural products far beyond anything yet discovered. In terms of mechanism of action, the amide linked *N*-methyl pyrroles in distamycin, netropsin and their relatives form curved structures that roughly match the curvature of the minor groove of DNA, and often with preferential affinity for AT-rich sequences. Crucially for the MTAID concept, these compounds do not target one cognate sequence, but are able to interact with many short, AT-rich DNA sequences, typically 5–10 base pairs in length. Binding to DNA, thereby, inhibits several DNA-centric functions and this is the major effect by which this class of compound acts [16]. There is definitive structural evidence for the binding of S-MGBs to the minor groove of DNA [17,18].

**Figure 2.**
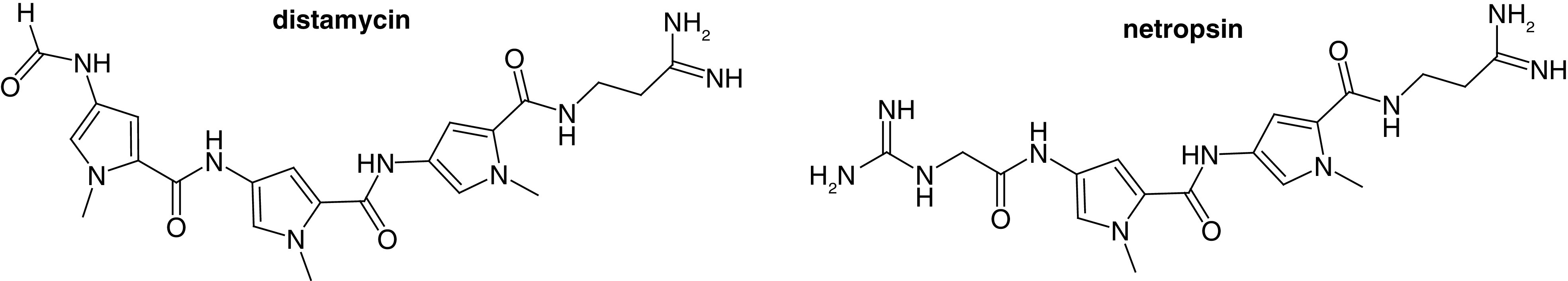
The structures of the natural products distamycin and netropsin.

Unsurprisingly, distamycin and netropsin themselves are not suitable for use as drugs because of toxicity and poor pharmaceutical properties. Their potential as starting points for the discovery of new drugs by structural modification is obvious and has been investigated by many groups apart from Strathclyde for anticancer and antibacterial applications in particular. In support of the minor groove of DNA as a biological target, other classes of compounds, most notably aromatic bis-amidines as antiparasitic compounds [19] and pyrrolobenzodiazepines as anticancer compounds [20] have been extensively developed. There are other approaches to obtain antibacterial activity that do not rely on single targeting, for example membrane disruptors [21–23]. The S-MGB program, however, is distinctive because, through different compounds of the same family, it has produced molecules with effectiveness in antibacterial, antifungal, antiparasitic and antiviral applications.

With regards to the template structure of distamycin, the S-MGB program has emphasized several structural changes to obtain its broad spectrum of activity: the amidine moiety has been replaced with less basic tertiary amines; different heterocycles have been investigated; the formyl ‘head group’ has been replaced with larger aromatics; and the amidine link to the ‘head group’ has been replaced with an amidine and an alkene (Figure 3). The potential to discover drugs across the range of prokaryotic and eukaryotic organisms and viruses is also exceptional. The following paragraphs outline the current state of research and development with respect to each type of pathogen.

**Figure 3.**
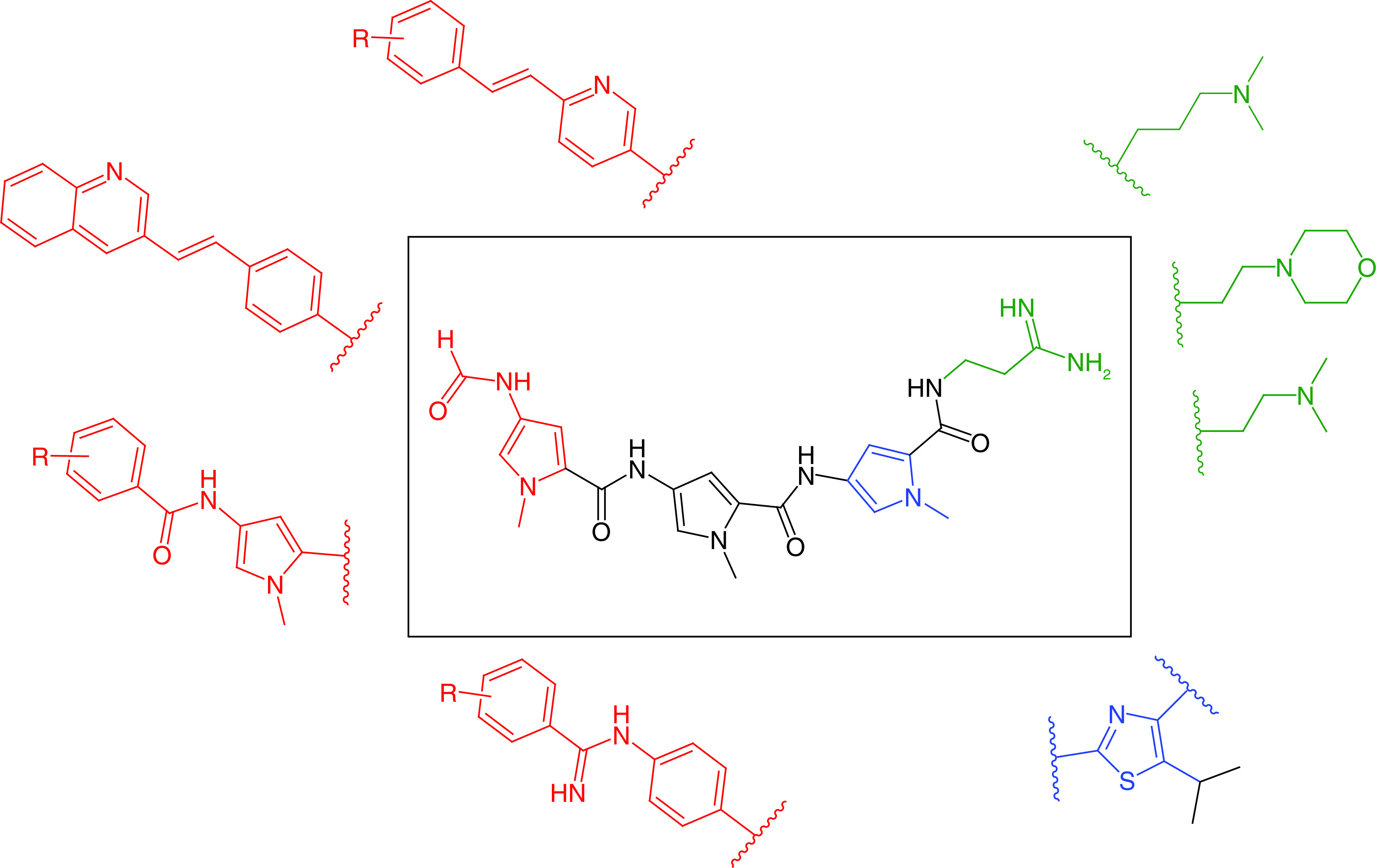
Exemplar structural modifications to the distamycin template that are seen in Strathclyde minor groove binders.

## Antibacterials

**Figure 4.**
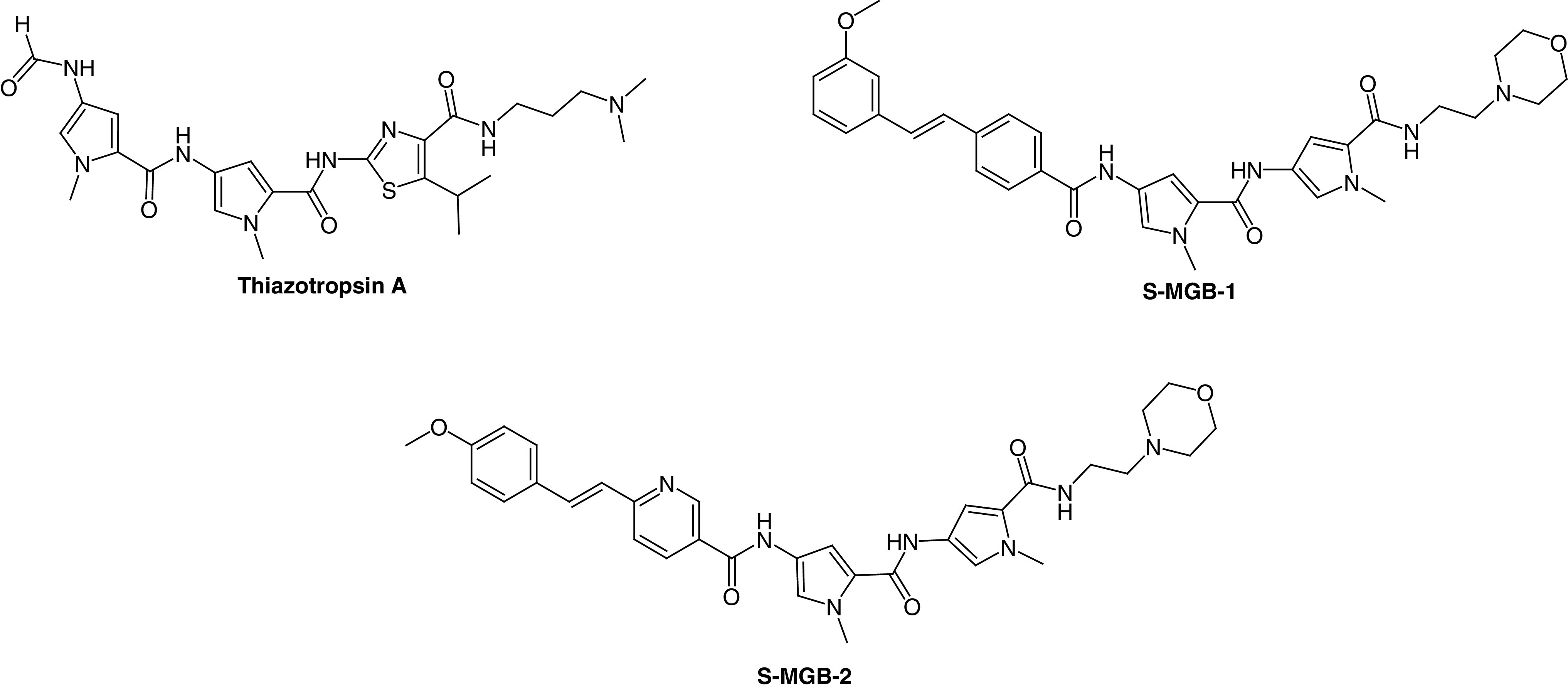
Structures of antibacterial Strathclyde minor groove binders.

**Figure 5.**
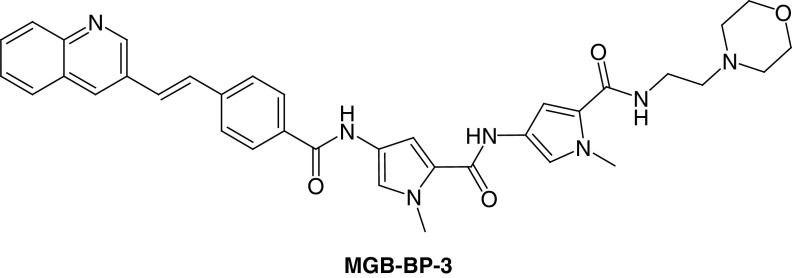
Structure of MGB-BP-3.

The first class of S-MGBs to be developed was for antibacterial activity. A design hypothesis for S-MGBs was put forward that suggested increasing the lipophilicity of the MGB in parts of the molecule that would make contact with the hydrophobic parts of the DNA surface, associated with the deoxyribose methylene group, for example. Some S-MGBs had larger *N*-alkyl groups than methyl but the modification that gave rise to the most active compounds in antibacterial and other activities was to replace the N-terminal amide with the isosteric but non polar alkene. The *N*-alkyl substitutions featured a *C*-isopropyl S-MGB, known as thiazotropsin A, that was used as a pilot compound to explore DNA binding preferences and physicochemical properties relevant to drug formulation such as self-aggregation (Figure 4) [24–26]. The alkene isosteric substitution led to a series of compounds with substantially improved activity against a wide range of pathogens such including Gram-positive bacteria, such as **S-MGB-1**, **S-MGB-2** and **MGB-BP-3** (Figures 4 & 5) [27].

Thiazotropsin A was one of the earliest S-MGBs to be investigated in detail. DNAase footprinting showed a pronounced preference for binding at the ACTAGC sequence. The explanation of this preference was shown by NMR of a short DNA oligomer containing ACTAGC complexed with thiazotropsin A. The thiazole formed an antiparallel 2:1 ligand:DNA complex with a strong hydrogen bond to G-NH_2_; thereby, accounting for the observed preference in the footprinting experiment [14]. Subsequent experiments with **S-MGB-2** and **MGB-BP-3** showed clear preferences for binding at AT rich sites such as ATATAT and AAATTT, again interpreted as antiparallel 2:1 complexes [27]; this preference will be seen below to be important in understanding the mechanism of antibacterial activity of **MGB-BP-3**. A property important for the formulation of an S-MGB medicine is self-association because of its effect on solubility and absorption. This was also investigated by NMR which showed for thiazotropsin A that it was associated into large aggregates in aqueous solution and that binding to DNA took place as disaggregated dimers [26,28]. Binding was shown to be largely enthalpically driven to compensate for entropy costs of disaggregation and conformational adjustments to the DNA duplex to accommodate the S-MGB. These properties fed into the development of **MGB-BP-3** and understanding its mechanism of action.

At Strathclyde, the first series of MGBs was screened for activity against a few bacteria and fungi, *Staphylococcus aureus, Escherichia coli, Aspergillus niger* and *Candida albicans.* While MIC_50_ values in the single digit micromolar range were observed, the advance to compounds of clinical significance came with the isosteric substitution of the alkene as found, for example, in **MGB-BP-3** [27]. This compound was the most active found against Gram-positive bacteria and together with a number of related MGBs, it was offered to a potential commercial partner, MGB Biopharma (Edinburgh, UK), which carried out preclinical and clinical development [5]. MGB Biopharma investigated more extensive activity profiles of the S-MGBs and selected **MGB-BP-3** for development with **S-MGB-2** as the back-up. In repeated assays over many years, **MGB-BP**-3 has an MIC_80_ value of less than 0.2 μM against *S. aureus*. **MGB-BP-3** and its close relatives; however, are only weakly active against Gram-negative bacteria.

The measured pK_a_ of **MGB-BP-3** is 5.26 making it a sparingly soluble molecule (<1 mg/ml) in aqueous solution at pH 7, a property associated with the weakly basic morpholino tail group and is poorly absorbed. Its properties made it suitable for treatment of GI infections such as caused by *C. difficile* and MGB Biopharma selected *C. difficile* associated disease as its clinical target. Preclinical development concentrated upon formulation and upon the effect of **MGB-BP-3** on infection in an animal model (hamster) of human disease; in this model the preferred GI release capsule formulation was found to be superior to the then ‘gold standard’ drug, vancomycin, both in speed of cure and in avoidance of recurrent infection. **MGB-BP-3** proceeded to a successful Phase I clinical trial (EudraCT 000489–73) in 2016 at Hammersmith Hospital (London) in which it was notable that it cleared the gut microflora of Gram-positive bacteria but left the Gram-negative load substantially unaffected. The Phase IIa trial was carried out at several sites in the USA and Canada (NCT03824795; 2019–2020) and was also very successful; sustained cures were found at a modest oral dose of 125 mg, twice daily and very importantly, without disease recurrence. The lack of recurrence is accounted for by the rapid kill of *C. difficile* by **MGB-BP-3** and its consequent repression of the sporulation that leads to recurrent disease. These properties give **MGB-BP-3** significant potential benefits over vancomycin and a Phase III head-to-head trial has been agreed with the US FDA [5].

The strong performance of **MGB-BP-3** so far is remarkable and it is the most advanced S-MGB currently in development. A limitation, however, of S-MGBs noted above is the lack of activity against Gram-negative bacteria. Various experiments together have pointed to the conclusion that S-MGBs do not readily accumulate in the cells of Gram-negative bacteria but when they can enter, they are active. Such phenomena are informatively shown in two ways. First, with respect to access, because of the diaryl alkene (stilbenoid) substructure in this group of S-MGBs, they are intrinsically fluorescent; it is therefore possible to detect cell entry by fluorescence microscopy. Entry into intact cells of *E. coli* does not occur but in spheroplasts, in which the outer cell wall has been removed with lysozyme, S-MGBs accumulate. Similar results are found when the cell envelope is compromised with the efflux pump inhibitor phenylarginine-β-naphthylamide or membrane permeabilizer polymyxin B nonapeptide (manuscript in preparation). Moving toward a therapeutic application for Gram-negative infections, it has been shown that a combination of ceftazidime and **MGB-BP-3** is able to kill a clinically resistant culture from a patient with a *Klebsiella pneumoniae* infection at clinically relevant concentrations [29]. The specific origin of this synergy is unknown, but it is likely due to weakening of the cell envelope by ceftazidime and allowing MGB-BP-3 to penetrate to its intracellular target. Further experiments of this type and further structural developments in S-MGBs will undoubtedly lead to potential treatments for Gram-negative diseases also.

**Figure 6.**
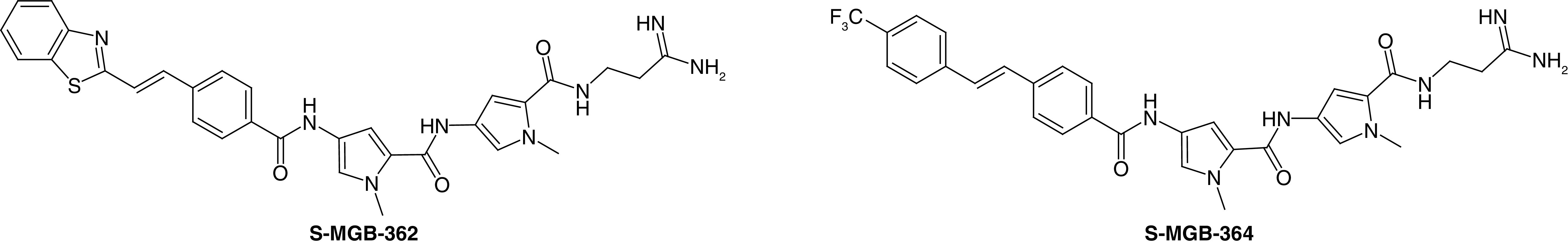
Structure of antimycobacterial Strathclyde minor groove binders.

Encouraging results have also been obtained for potential treatments for tuberculosis in partnership with the University of Cape Town [30]. Of the compounds evaluated **S-MGB-362** and **S-MGB-364** are good examples of highly significant outcomes (Figure 6). Both had strong activity against *Mycobacterium tuberculosis* HR-37-Gfp (MIC_99_ 0.39 and 1.56 μM respectively) but more importantly, the activity was maintained at significant levels in macrophages infected by the clinical strain HN878. These two S-MGBs have gone on to further study in South Africa and recent results show that **S-MGB-364** is active in an *in vivo* model of tuberculosis thus establishing proof of concept for S-MGBs as antituberculosis agents [31]. Interestingly with respect to the initiation of the study, the use of non-ionic surfactant vesicles ([NISV], niosomes) as delivery agents improved the activity of **S-MGB-362** and **S-MGB-364** by about twofold, a result that further supports the developability of these compounds.

In terms of their overall biological profile, the studies in South Africa have yielded further important information. Treatment of macrophages with **S-MGB-362**, **S-MGB-364** or **S-MGB-364**-NIV formulation was found not to cause DNA damage as shown by greatly reduced γ-H2Ax levels compared with that produced by the DNA harmful compounds, hydrogen peroxide and dimethylsulfoxide. Taken together the evidence shows that S-MGBs in this study do not damage DNA, impair metabolic function and viability of host cells. As noted above, **S-MGB-364** was the only active S-MGB *in vivo*. Intranasal administration of **S-MGB-362** (10 mg/kg) together with rifampicin had no effect on bacterial loads. On the other hand, both **S-MGB-364** and **S-MGB-364**-NIV formulation showed one log reduction in bacterial burden. Moreover, when in NIV formulation, reduced pathology and proinflammatory cytokine production (IL-1α, IL-17 and IFN-γ) was observed. It is interesting to note that S-MGBs with amidine tail groups have increasingly featured as lead compounds for several applications.

The preclinical and clinical studies of **MGB-BP-3** suggested a number of questions about its mechanism of action. Did it indeed bind to AT rich regions of DNA in treated bacteria? Were multiple biochemical events inhibited? Consequently, was there high resilience to the development of resistance? These questions have been addressed in RNA-Seq experiments using *S. aureus* as the model organism, which identified significant changes in 698 transcripts in the expression profile [32]. Levels of glycolysis-related enzymes were seen to increase and those of pentose phosphate pathway were reduced. Flux through the tricarboxylic acid cycle was also reduced due to a reduction in citrate synthase and isocitrate dehydrogenase levels. These changes are associated with energy depletion, which would be consistent with the rapid killing of *S. aureus* in culture, and the failure to enter a sporulation phase.

Taking observations to the level of control, DNase I and permanganate footprinting showed that binding to SigA promoters and inhibition of promoter isomerization by RNA polymerase holoenzyme took place. SigA promoters control essential functions in *S. aureus.* Specifically, **MGB-BP-3** was found to bind to the *dnaD* and *mraY* promoter regions; thereby, interfering with transcriptional initiation of these genes, which had been selected based on significant downregulation on drug challenge and quantitative reverse transcriptase (qRT)-PCR. Evidence for these binding sites came from DNAse and permanganate footprinting of the relevant regions of genomic DNA. None of the above precludes the possibility that DNA-centric events other than blocking transcription of essential genes contribute to the mechanism of action of S-MGBs.

At last turning to the most important question of the emergence of resistance, *S. aureus* was repeatedly challenged by **MGB-BP-3** in multiple passages. *S. aureus* was grown at sub-MIC_80_ concentrations of either **MGB-BP-3** or rifampicin as a control for 80 generations. Only resistance to rifampicin was observed. This is strong evidence for the resilience of **MGB-BP-3** to drug induced resistance. Taking all of the evidence together, **MGB-BP-3** can therefore be described by a profile that implies intrinsic resilience and avoidance of future bacterial resistance, a major potential benefit.

## Antiparasitics

**Figure 7.**
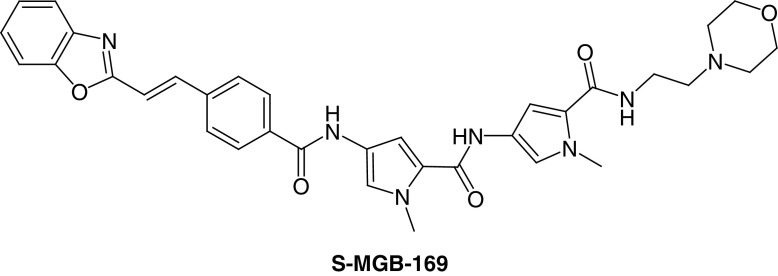
Structure of antimalarial Strathclyde minor groove binders.

The S-MGB project has benefited greatly from collaborations with outstanding biologists. Early in the project a panel of S-MGBs was speculatively screened by Professor Michael Barrett and Professor Harry de Koning (University of Glasgow) for activity against *Trypanosoma spp.*, following up some positive results at Strathclyde (Carol Clements). From these results not only was a substantial project into animal African trypanosomiasis (AAT) treatment established but also contact was made with Dr Vicky Avery (Griffith University, Australia) who was able to evaluate S-MGBs against *Plasmodium falciparum*. Of several highly active compounds, one, **S-MGB-169**, had exceptionally high antiplasmodial activity (EC_50_ = 38 nM) against the 3D7 strain and importantly was equally effective against the chloroquine resistant strain, Dd2, with an accompanying selectivity index compared with the human HEK cell line of greater than 500 (Figure 7). For reasons of human and financial resource limitation, this strong lead has not been further investigated [33].

**Figure 8.**
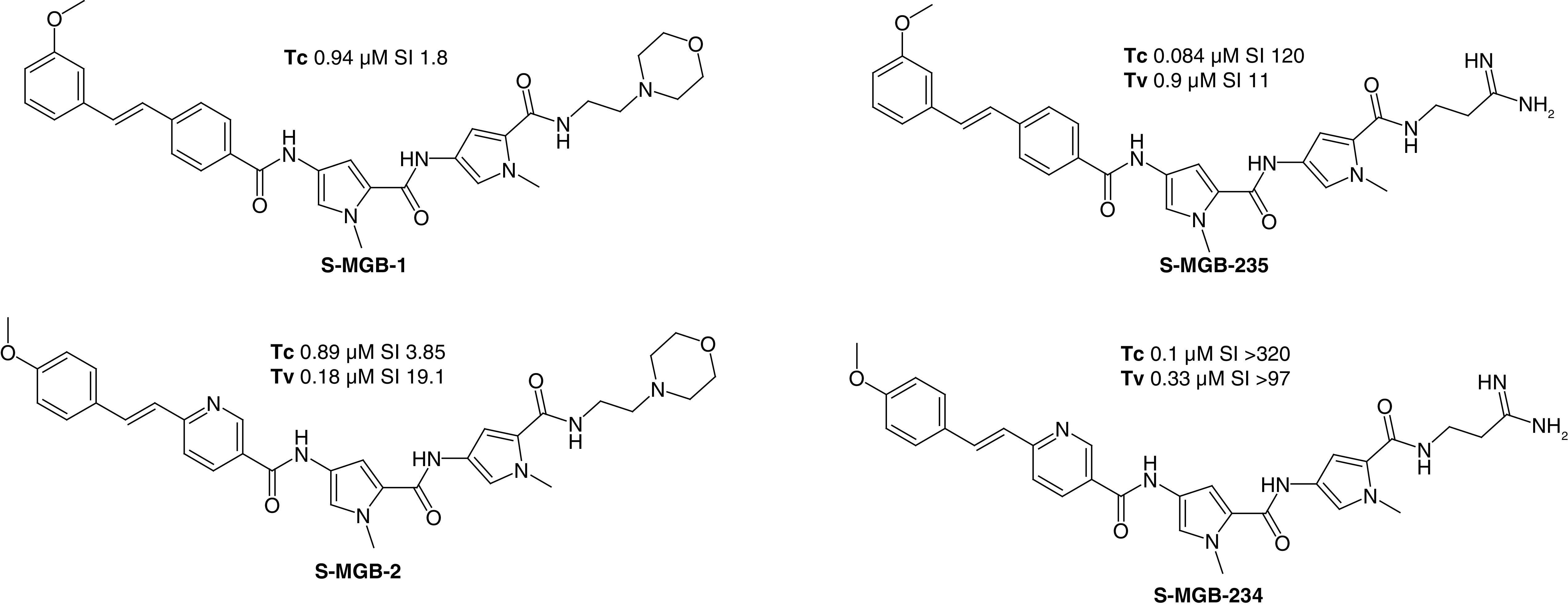
Structures and activities of antiparasitic Strathclyde minor groove binders. Tc: *Trypanosoma congolense* IC50; Tv: *Trypanosoma vivax* IC_50_; SI: Selectivity index, defined as HEK293T cell IC_50_ divided by Tc or Tv IC_50_.

Much more thoroughly studied have been antitrypanosomal compounds for AAT, several of which have been found to be curative in mouse models of trypanosomiasis, including both the species largely responsible for AAT, *T. congolense* and *T. vivax* [34]. Minor groove binders of another chemical class, the bis-amidines, are also known to be potent trypanocides [15,19]. At Strathclyde and Glasgow an iterative process of design and synthesis with screening *in vitro* led to the identification of active compounds sufficiently non toxic to mammalian cell lines (murine L6 and human HEK293T) to justify evaluation in *in vivo* models of disease. A general characteristic of the antitrypanosomal S-MGBs is the amidine tail group, which is permanently protonated at physiological pH in contrast to the weakly basic morpholine group of the developed antibacterial compound, **MGB-BP-3**; the amidine group gives both improved solubility and reduced toxicity presumably as a consequence of its much lower lipophilicity. As an example of the importance of this difference, the profiles of **S-MGB-1** and **S-MGB-235** can be compared. **S-MGB-235** is both ten-times more active against *T. congolense* and has a 100-fold better selectivity index; a similar relationship is found for **S-MGB-2** (morpholine) and **S-MGB-234** (amidine) (Figure 8). These properties contributed significantly to the selection of **S-MGB-234** and **S-MGB-235** as candidates for *in vivo* evaluation. Other important features were their activity against the diminazene-resistant *T. congolense* strain, DimR [34]. **S-MGB-235** was especially effective achieving a complete cure with greater than 60 days median survival in a group of four mice at an i.p. dose of 10 mg/kg twice, in comparison with 11 days survival for untreated mice.

The mechanism of action of typical diamidine minor groove binding trypanocidal drugs involves destruction of the kinetoplast and S-MGBs might be expected to do so too. A *T. b. brucei in vitro* generated isometamidium-resistant line with reduced mitochondrial membrane potential and lacking a kinetoplast, ISMR1, has been used to investigate this phenomenon for S-MGBs. **S-MGB-234** was effective against this mutant showing that the kinetoplast is not essential for activity and that the kinetoplast, which is destroyed by diamidine exposure, is not the main target of the S-MGBs. A further distinction from conventional diamidine minor groove binders was found in that **S-MGB-234** (and other S-MGBs) retained activity against *T. brucei* lines resistant to diamidines (specifically because they lack the transporters *Tb*AT1/P2 and HAPT/AQP2). Therefore S-MGBs have routes of uptake other than those involved in diamidine internalization. There was no evidence that DNA replication inhibition was the main mode of action of S-MGBs but, as was noted for antibacterial S-MGBs, there are many DNA-centric candidates for lethality. A pathology observed using S-MGBs; however, was segregation and their morphology aberrations in DNA-containing organelles upon treatment of *T. b. brucei* with **S-MGB-234**. Cells with multiple kinetoplasts and nuclei (MKMN) were observed to accumulate over time indicating problems with cytokinesis. The discovery of curative S-MGBs effective against both pathological species of trypanosome in AAT is something unique to the S-MGB class. Moreover, the activity is retained against strains resistant to currently used drugs and the lack of the emergence of resistance has been demonstrated in laboratory tests [34]. Further work has identified other S-MGBs with improved profiles and work continues to expand proof of concept to other trypanosomes and genera of parasites.

## Antifungal applications

**Figure 9.**
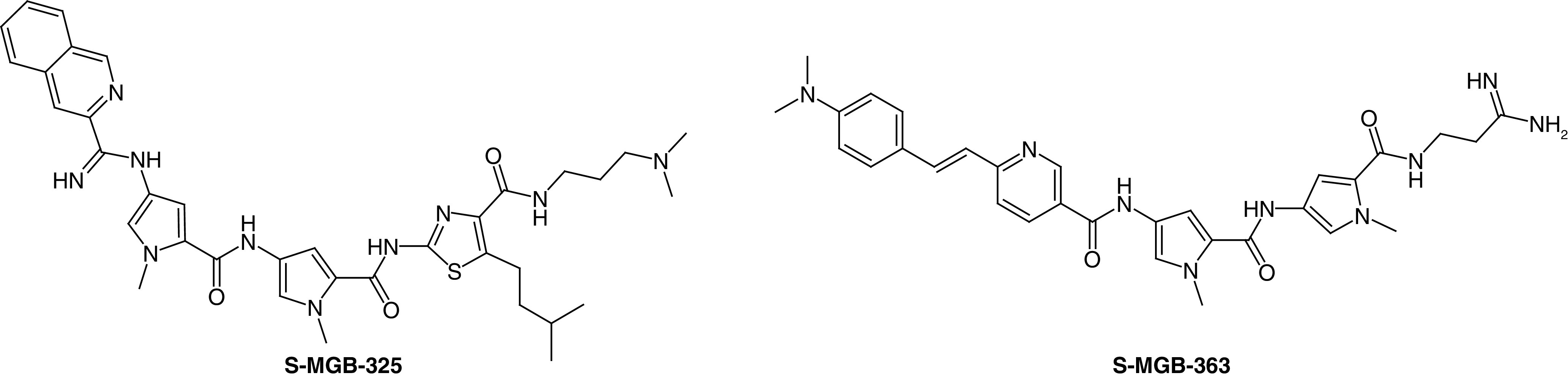
Structure of antifungal Strathclyde minor groove binders.

Before the identification of the antibacterial properties of **MGB-BP-3** it was thought that an antifungal application might be the first to be developed. It was not until a substantial panel of S-MGBs was screened against several fungal species by the Community for Open Antimicrobial Drug Discovery that the full potential of S-MGBs in antifungal applications became clear [35]. Some of the new aspects of S-MGB biology associated with fungi could already be gleaned from a previous limited study at Strathclyde in which the composition of the bacterial cell wall appeared to be critical for activity. **S-MGB-325** was very active against *Cryptococcus neoformans* (MIC_70_ 0.25 mg/l) but inactive against *Candida albicans* (Figure 9). A plausible explanation for this difference is based upon the cell wall structure of the two fungi. The outer chain mannans of *C. albicans* contain negatively charged phosphodiester links which are absent in *C. neoformans*. Consequently, the phosphodiester anion could sequester dicationic MGBs, preventing uptake into the cell and causing the lack of activity [36].

Following the preliminary investigation of antifungal S-MGBs, we have embarked on a specific program of antifungal drug discovery with Professor Michael Bromley at the University of Manchester. This led to the discovery of **S-MGB-363**, which is of interest from a number of points of view. First, the screening results show that it has a broad activity spectrum including both animal and plant pathogens with levels of clinical significance (between 0.8 and 6.25 mg/l) for *Rhizopus arrhizus, Fusarium oxysporum, Aspergillus flavus, Scedosporium prolificans, Candida albicans, Cryptococcus neoformans, Candida glabrata, Aspergillus fumigatus* and *Candida auris*. Second, there is activity against the emerging resistant pathogen, *C. auris* [37].

With regard to mechanism of action, **S-MGB-363** lends itself to direct study because it is intrinsically highly fluorescent. This property has made it possible to demonstrate that the compound first associates with the fungal cell wall before accumulating in the nucleus. The latter is firmly in line with the proposed mechanism of action of a DNA binding compound.

Based on the preliminary success with **S-MGB-363**, a full drug development campaign is currently underway.

## Antiviral prospects

**Figure 10.**
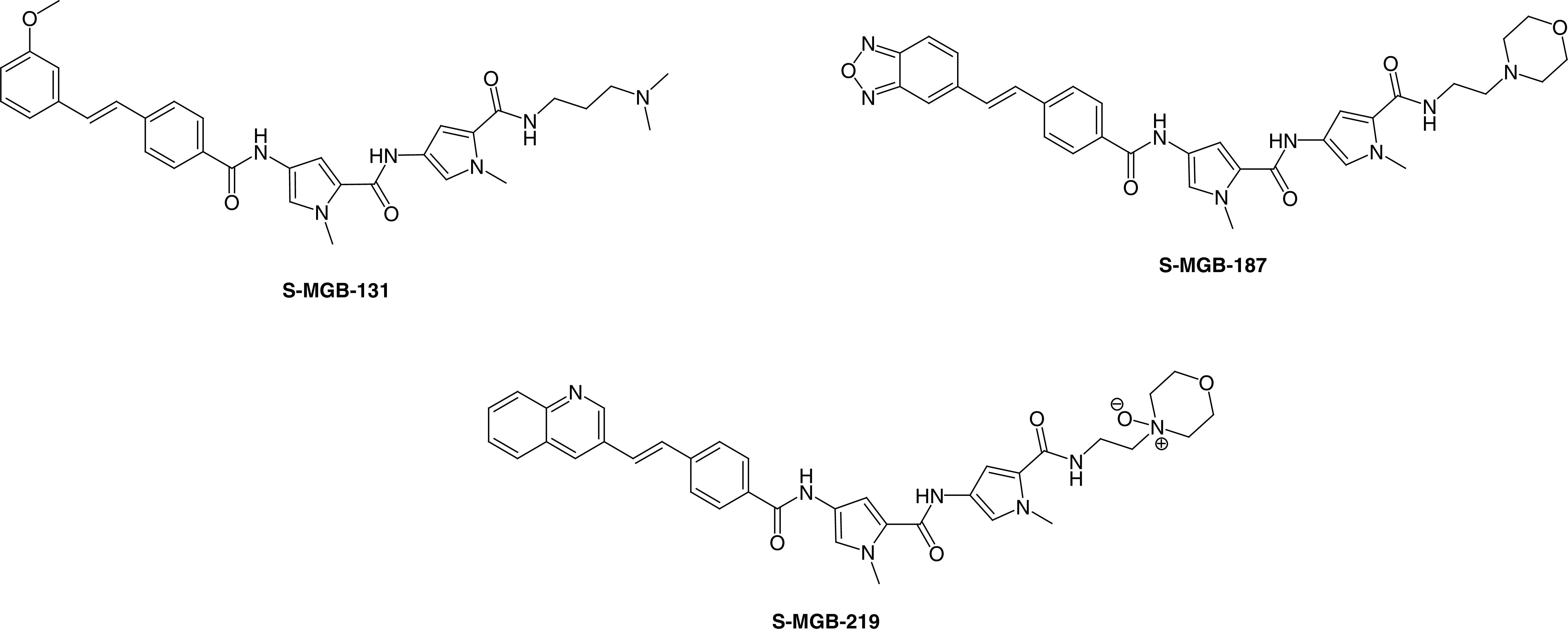
Structures of antiviral Strathclyde minor groove binders.

While not immediately obvious that S-MGBs would be effective against viral infections, in particular against RNA viruses, it would be remiss in the current pandemic not to look into the possibility. Significant evidence for antiviral activity of S-MGBs was obtained some years ago in collaboration with Professor Arvind Patel (University of Glasgow) against HCV [unpublished results]. HCV is a negative ssRNA virus and would therefore not intuitively be considered a therapeutic target for DNA minor groove binders. It was, however, an important target at the time of the investigation and important initial data were obtained. The most significant compounds were **S-MGB-131**, **S-MGB-187** and **S-MGB-219**, all of which had subnanomolar IC_50_s for the prevention of viral growth with selectivity indices greater than 1500 for the host cell (Figure 10).

The significant activity against an RNA virus raised the interesting question of whether S-MGBs bind to RNA. There is substantial evidence that other types of DNA minor groove binder; classical minor groove binders such as DAPI and Hoechst 33258 and bis-amidines bind to RNA [38,39]. It is therefore reasonable to expect that S-MGBs do so too. This has been shown to be the case by using fluorescence intercalator displacement assays with the double-stranded homopolymer, polyadenylic acid-polyuridylic acid, as an exemplar dsRNA target. Many, if not most S-MGBs, have been found to bind to dsRNA in these assays [40]. Bearing in mind the structural complexity of RNA it is possible that DNA minor groove binders, including S-MGBs, could form many different types of complex with RNA. In light of this new information, it is necessary to acknowledge the potential inadequacy of the term ‘S-MGB’. Recognizing that the range of targets engaged by these compounds includes both DNA and RNA, a more appropriate designation would be Strathclyde nucleic acid binders (SNAB).

Antiviral activity is of great importance in the context of the current pandemic and a selection of SNABs (formerly, S-MGBs) was evaluated *in vitro* at Public Health England. The laboratory test assessment of efficacy of S-MGBs against SARS-CoV-2 resulted in three ‘hit’ compounds with activities between 5 and 10 μM, which were low enough for progression to animal studies. Of these, **S-MGB-363** was chosen based on existing data that demonstrated that it was well-tolerated in animal (mouse experiments), and that it localized substantially in lung tissue when administered by injection (Figure 9). In the hamster model of COVID-19 at Public Health England **S-MGB-363** reduced viral load in the hamster and also reduced the damage that the virus caused to the lungs and nasal cavity; thereby, establishing proof of concept *in vivo* for the antiviral activity of SNABs [40].

## Conclusion

The family of antibiotic molecules, known as Strathclyde nucleic acid binders (SNABs), have a mode of action involving binding to DNA or RNA in the target infectious agents. They bind at multiple loci on nucleic acids, with the result that mutation to confer resistance via target engagement modification is unlikely. This renders the SNAB family of molecules resilient to generating resistance. Different members of the family have been shown in vitro and in vivo to have efficacy as anti-bacterials, anti-parasitics, anti-fungals, and anti-virals. A lead antibacterial, MGB-BP-3, has successfully completed phase II clinical trials to treat C. difficile infections and is ‘phase III ready’. The SNAB family offers great potential in our fight against anti-microbial resistant infections.

## Future perspective

Any treatment with a DNA binding drug requires confidence that there is no resulting genotoxicity. Evidence accumulating over the past 20 years from studies in many laboratories of many compounds that bind non covalently in the minor groove show that there is no class liability for DNA damage [41,42]. It is also the case that not every SNAB binds to every nucleic acid target or, importantly, reaches targets in cells, especially host cells. There is a firm basis for species cellular selectivity emerging, which gives confidence for the exploitation of non covalently binding minor groove binders in therapeutics. In dealing with most infections, courses of treatment are short, a fact that also mitigates the risk of chronic damage to DNA. The essence of SNAB activity, therefore, is selective location at pathogen nucleic acids and consequent lethal disruption of several essential biological mechanisms of the pathogen.

One part of the original concept for S-MGBs was that their multitargeted activity through DNA-centric biological processes would yield compounds with activity against a wide range of infectious agents, and this remains true for SNABs. This has proved to be the case in practice. Activity has been found at therapeutically significant levels *in vitro* against Gram-positive bacteria, mycobacteria, parasites, fungi and viruses. In all cases, proof of concept activity in animal models of relevant disease has been found with different compounds characteristically active against different infectious agents. One SNAB has progressed successfully through a Phase IIa clinical trial for the treatment of *C. difficile* infections. Serious therapeutic challenges such as TB and new fungal infections (*C. auris*) can in principle be met using appropriate S-MGBs.

Underlying the results supporting development of SNABs for infectious diseases is evidence for the second and most important part of their therapeutic profile, namely resilience to the emergence of resistance. This also was part of the original concept for S-MGBs. Thus, evidence from studies of *S. aureus* and *T. spp.* with different SNABs is consistent with their inhibiting several biological functions. Resistance has not been found when sought in multiple passage experiments with *S. aureus* and *T. congolense* as the target organisms [29,31]. With respect to antitrypanosmal activity, the SNABs are probably the only series of compounds to show significant activity across a wide range of disease-causing species including *T. brucei, T. congolense, T. vivax* and *T. cruzi.* Indeed, SNABs are perhaps the only class of compound to show significant activity against pathogens from biologically distinct families: bacteria, parasites, fungi and viruses. One of the most surprising and potentially significant discoveries is the activity of SNABs against RNA viruses, including SARS-CoV-2. These results open a new field of research.

In summary, SNABs, formerly S-MGBs, have been shown experimentally and in the clinic to have exceptional potential as anti-infective drugs working in a manner consistent with their original design concept.

**Executive summary**

- Many of the resistance problems associated with current anti-infective drugs are a result of their single-target design.
- Multitargeted anti-infective drugs are likely to be superior to single biological target drugs due to their lower susceptibility to target-related resistance mechanisms.
- Strathclyde minor groove binders, now termed Strathclyde nucleic acid binders (SNABs), are a class of compound that have been designed based on the multitargeted anti-infective drugs principle.
- SNABs have demonstrated clinically relevant antibacterial properties, namely in the compound, **MGB-BP-3**, which has completed Phase IIa clinical trial for the treatment of *C. difficile* associated disease.
- SNABs have also demonstrated *in vivo* animal model efficacy against mycobacteria, trypanosomes and fungi.
- In the antiviral space, SNABs have demonstrate a positive effect in *in vivo* animal models of SARS-CoV-2.
